# Stabilization of golden cages by encapsulation of a single transition metal atom

**DOI:** 10.1098/rsos.171019

**Published:** 2018-01-03

**Authors:** Hui-Fang Li, Huai-Qian Wang

**Affiliations:** College of Engineering, Huaqiao University, Quanzhou, 362021, People's Republic of China

**Keywords:** nanocluster, structure, density-functional theory, stability, photoelectron spectra

## Abstract

Golden cage-doped nanoclusters have attracted great attention in the past decade due to their remarkable electronic, optical and catalytic properties. However, the structures of large golden cage doped with Mo and Tc are still not well known because of the challenges in global structural searches. Here, we report anionic and neutral golden cage doped with a transition metal atom MAu_16_ (M = Mo and Tc) using Saunders ‘Kick' stochastic automation search method associated with density-functional theory (DFT) calculation (SK-DFT). The geometric structures and electronic properties of the doped clusters, MAu_16_*^q^* (M = Mo and Tc; *q* = 0 and −1), are investigated by means of DFT theoretical calculations. Our calculations confirm that the 4d transition metals Mo and Tc can be stably encapsulated in the Au_16_^−^ cage, forming three different configurations, i.e. endohedral cages, planar structures and exohedral derivatives. The ground-state structures of endohedral cages *C*_2v_ Mo@Au_16_^−^-(a) and C_1_ Tc@Au_16_^−^-(b) exhibit a marked stability, as judged by their high binding energy per atom (greater than 2.46 eV), doping energy (0.29 eV) as well as a large HOMO–LUMO gap (greater than 0.40 eV). The predicted photoelectron spectra should aid in future experimental characterization of MAu_16_^−^ (M = Mo and Tc).

## 1. Introduction

Nanoclusters display many new properties, which are usually not found in their bulk counterparts [1–3]. These novel properties can be attributed almost to strong relativistic effects and finite-size quantum effects [4,5]. Gold clusters, in particular, have received special attention due to their potential technological applications as in the fabrication of materials in catalysis [6–11], chemical/biological sensors [12], medical sciences [13] and so forth. As the geometry of the cluster is closely related to its properties, an understanding of the cluster geometry is of primary interest. It is very important to identify their geometric structures for the controlled use of clusters in future nanotechnology. In the past couple of decades, both gold clusters and gold cluster doping with impurity atoms of alkali metal or transition metal have attracted the attention of theoreticians and experimentalists working in the field of cluster science [14–26]. The results of the previous investigations indicate that the introduction of a transition metal dopant atom in the gold cluster can change its structure and electromagnetic properties significantly [22–24]. In particular, photoelectron spectroscopy (PES) in combination with density-functional theory (DFT) calculations [27] revealed that the ground-state structure of the Au_16_ cluster anion has a highly stable hollow cage with a large internal volume similar to that of fullerenes [28]. This feature leads to the possibility of forming a new class of golden cages with particular properties by endohedral doping similar to those of the endohedral fullerenes. Subsequently, the investigation of doping one guest atom into the Au_16_^−^ cage has prompted immediate extensive interest by both theorists and experimentalists with purpose to design novel endohedral gold-caged clusters, as the chemical and physical versatility can be exploited by tuning the structural and electronic properties of gold clusters [29–34]. The most recent *ab initio* calculations showed the gold-covered bimetallic clusters M@Au*_n_* (*n *= 8–17) with closed-shell structures obeying the 18-electron rule and starting from *n *= 9 the doped-metal atom prefers to be entirely covered by pure gold atoms to form the lowest energy structure [29]. Subsequently, a series of doped gold anion clusters MAu_16_^−^ (Ag, Zn, In and Cu) have been systematically studied using PES experiment and theoretical calculations by Wang *et al.* [18,19]. It is found that Ag, Zn and In can all be doped inside the Au_16_^−^ cage with little structural distortion. Similar to Cu, they transfer their valence electrons to the golden cage and form endohedral charge transfer complexes. However, in contrast to a previous theoretical prediction on MAu_16_^−^ (Ag, Zn, In and Cu), the doping Au_16_^−^ cluster with a Si, Ge or Sn atom led to completely different structures, forming exohedral structure where the tetrahedral golden cage is completely distorted due to the strong M–Au local interactions [30]. Many physical chemists have made many efforts to deal with the global optimization of clusters using automated procedures [35–38]. For example, Car & Parrinello's [35] well-known ‘dynamic simulated annealing' combines molecular dynamics (MD) and DFT. Shayeghi *et al.* [36] present an approach for the global optimization of monoatomic or binary clusters. Very recently, a global optimization technique, using neural network potentials combined with the basin-hopping method, to study medium-sized metal clusters was proposed by Jiang *et al.* [39,40].

Up to now, Au_16_ cluster and golden cage Au_16_ doping with impurity atoms of 3d transition metal and alkali metal have attracted the attention of researchers in both theoretical and experimental studies devoting themselves to working in cluster science [16,18–23]. In a recent study, we have provided the first theoretical evidence of endohedral doping of the golden cages by the early 4d transition metals Y, Zr and Nb in Au_16_^−^ cage [24]. The closeness of the vertical/adiabatic detachment energy (ADE) for doped clusters MAu_16_^−^ (3.506/3.632 eV for Y-doped, 2.693/2.837 eV for Zr-doped and 3.406/3.496 eV for Nb-doped) reveals a negligible geometry change between the anionic and neutral species. However, to the best of our knowledge, only a limited literature existed on the 4d transition metal impurity atoms as dopants in golden cage Au_16_ clusters, there are still many open questions for 4d transition metal atoms, Mo, Tc and so forth. For example, there is still no experimental evidence for verifying the predicted structures directly. What are equilibrium structures and relative energies for doped golden cage Au_16_ clusters? Are there preferred endohedral or exohedral doping nanostructures and how are these geometric structures formed? The large empty space inside the Au_16_ cluster anion cage allows for possible endohedral doping to form a new class of endohedral golden cages. The endohedral golden cages could display new chemical physical and catalytic properties which are different from those of the bare golden cage clusters. Thus, an accurate first-principles calculation based on the density-functional theory is fundamental to understand the structural and electronic properties of those clusters. Furthermore, most previous theoretical calculations on atomic clusters were building structures manually as well as the presumed symmetric constraints. An unconstrained global search on the cluster potential energy surface is needed.

In the present work, we report a theoretical study of doping a 4d transition metal atom M (M = Mo and Tc) into the Au_16_ cage cluster. A number of anionic and neutral doped golden cage isomers are obtained using Saunders ‘Kick' global search technique [41] combined with DFT calculation (SK-DFT). Recently, we have successfully employed the SK-DFT method for global minimum searches of relatively small clusters, and provided a comprehensive analysis of the ability of current methods to determine the geometry of the ground state of clusters [42–46]. The specific objectives of this work are fourfold: (1) to identify structures of the lowest-energy/low-lying clusters using a global optimization method coupled with DFT calculation; (2) to provide useful information for MAu_16_ cluster systems in future photoelectron spectroscopy experiment; (3) to compare the results of our extensive computations performed using SK-DFT with previously experimental findings on the host golden cage and some other 3d and early 4d transition metal atoms-doped gold clusters; (4) to characterize the stability of the lowest-energy clusters by computing their binding energy per atom, doping energy and the highest occupied and lowest unoccupied molecular orbit (HOMO–LUMO) gap. At this stage, although other energetically more favourable structures could not be ruled out strictly, we believe that the lowest-energy structures of MAu_16_^q^ found here are at least powerful candidates for their ground states, which are hoped to be verified in the future photoelectron spectroscopy experiments and calculations at more accurate levels of theory. This work should be interesting for future material physicists and chemists, especially those designing new materials.

## 2. Computational methods

The structure prediction of MAu_16_*^q^* (M = Mo and Tc; *q* = 0, –1) clusters is based on the Saunders ‘Kick' stochastic automation search method [41] combined with density-functional theory calculation (SK-DFT) which has been successfully applied in the structural prediction of a number of cluster systems [22–24,42–46]. All the mixed atoms, including 16 gold atoms and a single transition metal atom (Mo and Tc), are placed at the same point initially and then are ‘kicked' randomly within a size-controlled hollow sphere with a radius *R* for avoiding biasing search. The kick size (radius *R*) in the hollow sphere is 15 Å in this work. The kick method runs at the PBEPBE/LANL2DZ (‘PBEPBE’ functional [47] with a scalar relativistic effective core potential (RECP) and LANL2DZ basis set [48]) level up to 800 times until no new minima appeared. Afterwards, the top several isomers approximately 0.3 eV from each minimum at the PBEPBE/LANL2DZ level were all regarded as potential candidate lowest-energy structures to be further reoptimized and evaluated with the larger basis set. As no symmetry constraints are imposed, the geometries obtained should correspond to minima. The reoptimization and evaluation used PBEPBE exchange-correlation functional with the large basis set Au/SDD+2*f*/M/ECP28MWB, followed by vibrational frequency calculations. Here, ‘SDD+2f' denotes the Stuttgart/Dresden RECP valence basis [49,50] augmented by two sets of *f* polarization functions (exponents = 1.425, 0.468) for Au, and ‘ECP28MWB' denotes the Stuttgart contracted pseudo-potential basis set for 4d transition metal atom M (M = Mo and Tc) [51,52]. All calculations were performed using the Gaussian 09 package [53].

**Figure 1.**
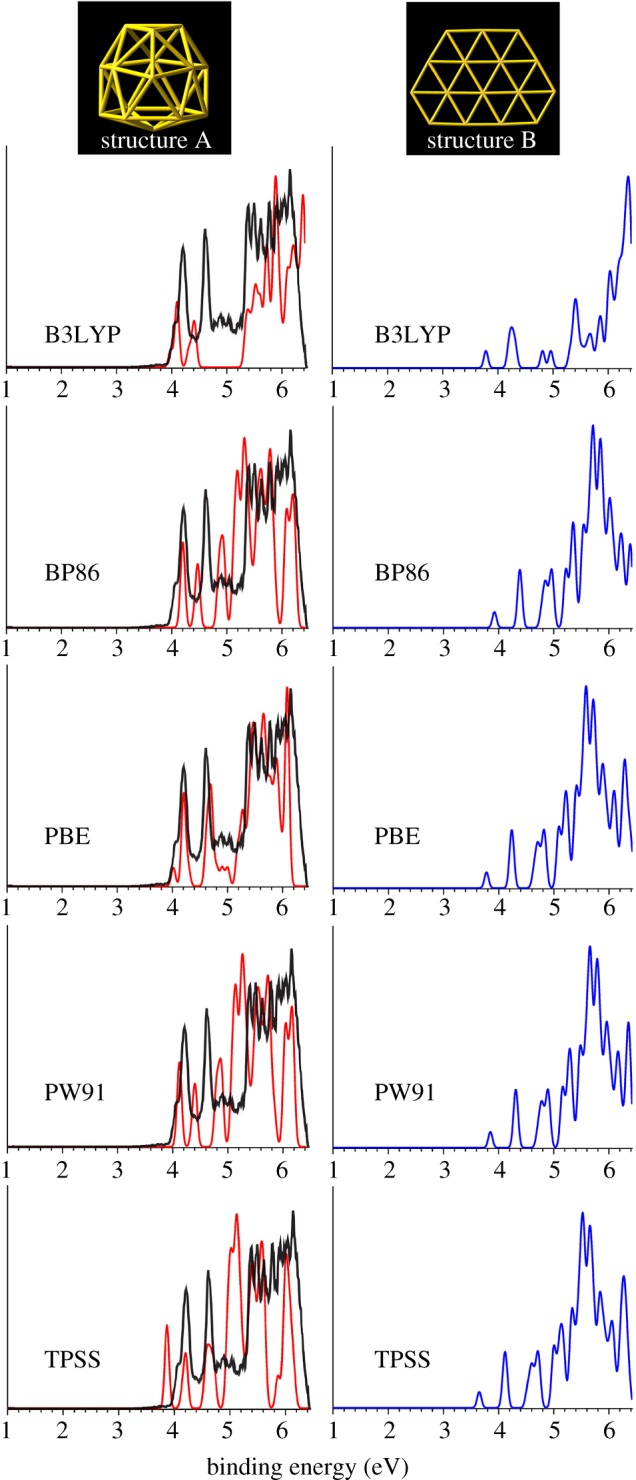
Structures and simulated photoelectron spectra for the two low-lying isomers of Au_16_^−^ using five different methods. For comparison, the experimental spectra of Au_16_^−^ cage are shown by the black curves. The experimental PES spectra are cited from [27].

The accuracy of PBEPBE/Au/SDD+2*f*/M/ECP28MWB level of theory was validated using five exchange-correlation functionals (PBEPBE [47], B3LYP [54], BP86 [55,56], PW91 [57] and TPSS [58]) with the same RECP valence basis SDD+2*f* on pure gold clusters Au_16_*^q^* (*q* = 0, –1). The first ADE and vertical detachment energy (VDE) are calculated and photoelectron spectra are also simulated. Furthermore, to quantitatively compare simulated spectrum with the experimental spectrum [18,27], we calculate the root-mean-square deviation (RMSD) for the labelled peaks X ∼ C [18]. Comparing the calculated first ADE/VDE, RMSD and simulated photoelectron spectra with measured results by PES experiment for the Au_16_^−^, PBEPBE/SDD + 2*f* level of theory gives very good agreement with the experimental observations (table 1 and figure 1), and, therefore, the same level has been selected as the method of choice for MAu_16_*^q^* (Mo and Tc; *q* = 0, –1) species also. Here, the first ADE is determined by calculating the energy difference between the optimized anion geometry and the optimized neutral geometry. The first VDE is defined as the energy difference between the neutral clusters at optimized anion geometry clusters and optimized anion clusters. Then, the first VDE is added to the orbital energies of the deeper occupied orbitals to obtain VDEs of the higher detachment channels. The VDEs so obtained are fitted with a full width at half-maximum (FWHM) of 0.09 eV to yield the simulated spectra, which are used to compare with the experimental spectra. This method has been used successfully in a number of previous studies and has been shown to yield VDEs in good agreement with experimental photoelectron spectra [59–64].

**Table 1.** Comparison of structural and energetic characteristics of structures A/B using different methods for pure neutral and anionic Au_16_.*^a^*

method	charge	SM	Sym	*E*_T_	*ΔE*	ADE	VDE	RMSD
structure A								
B3LYP	–1	2	*D*_2d_	−2173.21564633	0.000	3.83	3.98	0.13
	0	1	*S*_4_	−2173.07498251	0.000			
BP86	–1	2	*S*_4_	−2174.86868788	0.000	4.05	4.18	0.23
	0	1	*C*_1_	−2174.71981703	0.000			
**PBE**	–**1**	**2**	***D***_**2d**_	−**2173**.**23223352**	**0**.**000**	**3**.**89**	**4.02**	**0.06**
	0	1	*D*_2d_	−2173.08939878	0.000			
PW91	–1	2	*C*_2_	−2174.24031705	0.000	3.96	4.10	0.19
	0	1	*D*_2d_	−2174.09458239	0.000			
TPSS	–1	2	*C*_1_	−2172.11831722	0.000	3.65	3.86	0.20
	0	1	*S*_4_	−2171.98436357	0.000			
structure B								
B3LYP	–1	2	*C*_2v_	−2173.23596465	−0.553	3.74	3.79	0.26
	0	1	*C*_2v_	−2173.09846579	−0.639			
BP86	–1	2	*C*_2v_	−2174.87590664	−0.196	3.89	3.93	0.25
	0	1	*C*_2v_	−2174.73275679	−0.352			
PBE	–1	2	*C*_2v_	−2173.23379128	−0.042	3.75	3.78	0.31
	0	1	*C*_2v_	−2173.09605333	−0.181			
PW91	–1	2	*C*_2v_	−2174.24073219	−0.011	3.82	3.86	0.28
	0	1	*C*_2v_	−2174.10028040	−0.155			
TPSS	–1	2	*C*_2v_	−2172.10762424	0.291	3.62	3.65	0.39
	0	1	*C*_2v_	−2171.97454872	0.267			
Expt.*^b^*						3.99 ± 0.03	4.03 ± 0.03	0.00

*^a^*Shown are the spin multiplicity (SM), symmetry type (Sym), total energy (*E*_T_, a.u.), the relative energy (Δ*E*, eV), calculated first adiabatic/vertical detachment energy (ADE/VDE, eV), and the RMSD (eV).

*^b^*References [18] and [27].

## 3. Results and discussion

**Figure 2.**
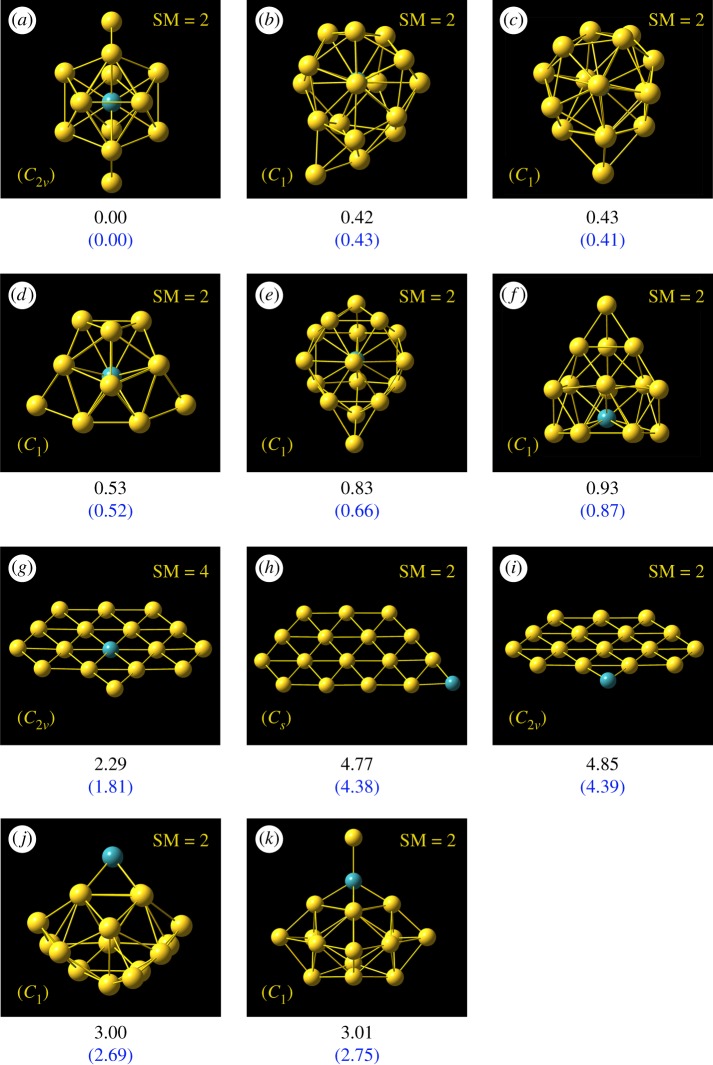
The 11 lowest-energy isomers of MoAu_16_^−^ cluster obtained by SK-DFT. All the energy values (in eV) given beneath each isomer are the relative energy with respect to the leading lowest-energy isomer. The relative energies in the first line are based on the PBEPBE/LANL2DZ level. The energy values in parentheses are based on the PBEPBE/Au/SDD+2*f*/M/ECP28MWB level of theory.

**Figure 3.**
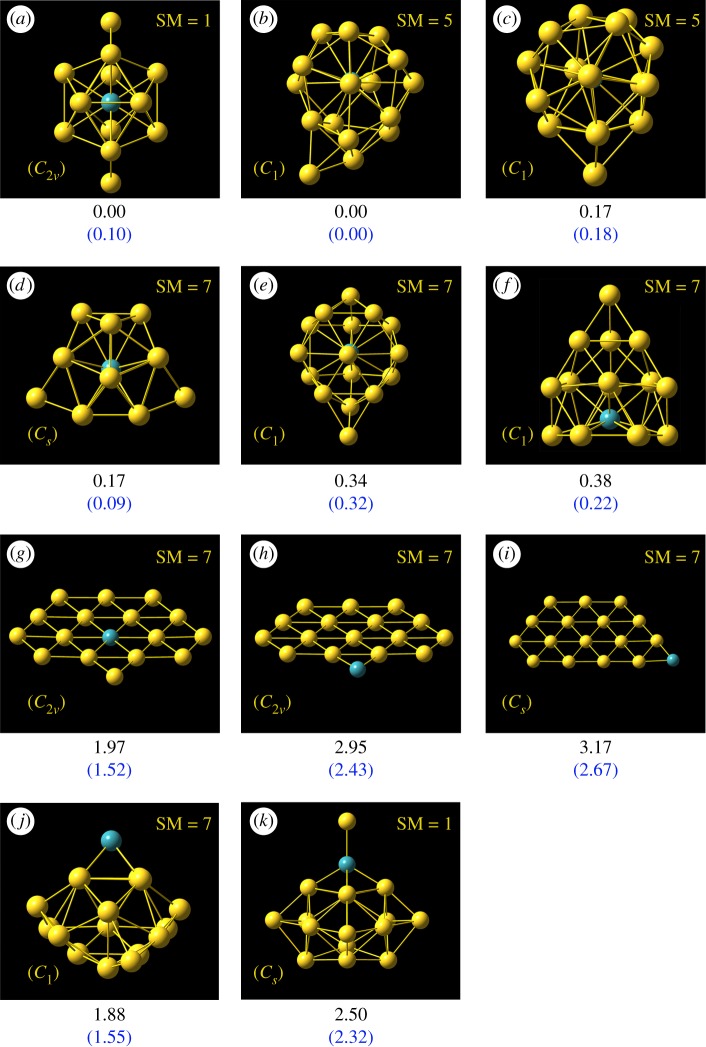
The 11 lowest-energy isomers of TcAu_16_^−^ cluster obtained by SK-DFT. All the energy values (in eV) given beneath each isomer are the relative energy with respect to the leading lowest-energy isomer. The relative energies in the first line are based on the PBEPBE/LANL2DZ level. The energy values in parentheses are based on the PBEPBE/Au/SDD+2*f*/M/ECP28MWB level of theory.

**Figure 4.**
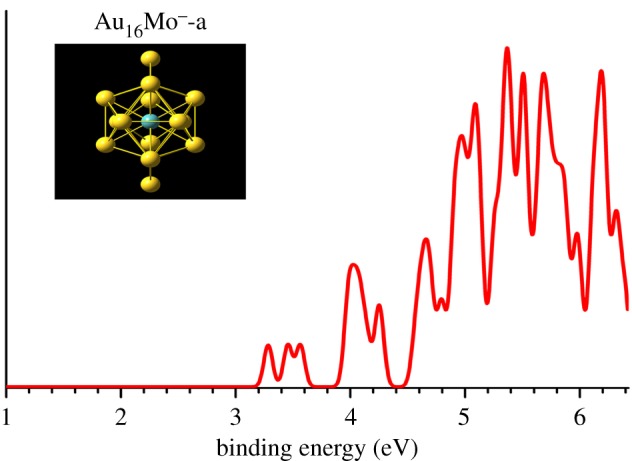
Structure and simulated photoelectron spectrum from the lowest-energy isomer of Mo@Au_16_^−^.

**Figure 5.**
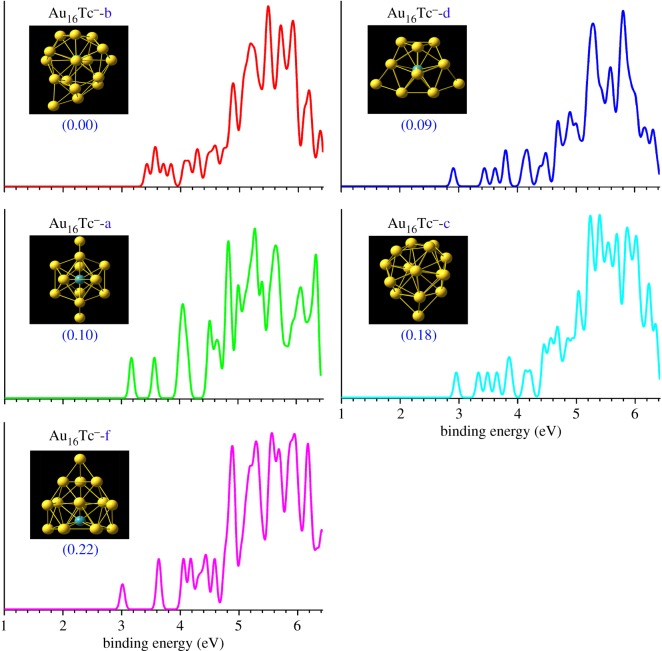
Structures, relative energies (Δ*E*) in eV and simulated photoelectron spectra from the top-five lowest-energy isomers (approximately 0.3 eV) of Tc@Au_16_^−^.

Two important types of structures A/B, simulated photoelectron spectra of Au_16_^−^ for structures A/B using five different functionals (B3LYP, BP86, PBE, PW91 and TPSS) in comparison with experimental photoelectron spectra [27] for host gold cluster Au_16_^−^ are shown in figure 1. Table 1 gives various structural and energetic characteristics of the top-two structures of host pure neutral and anionic Au_16_ clusters, including the spin multiplicity (SM), symmetry type (Sym), total energy (*E*_T_), the relative energy (Δ*E*), calculated/experimental first ADE/VDE and RMSD. The top-11 lowest-energy isomers of MAu_16_^−^ (M = Mo and Tc) obtained from a SK global search combined with DFT optimization and total energy calculation at the PBEPBE/LANL2DZ and PBEPBE/Au/SDD+2*f*/M/ECP28MWB levels are presented in figures 2 and 3 (the Cartesian coordinates of these low-lying isomers can be found in the electronic supplementary material). The energy values (in eV) given beneath each isomer are the relative energy with respect to the leading lowest-energy isomer. The energy values in the first line are based on the PBEPBE/LANL2DZ level. The energy values in parentheses in blue are calculated at the PBEPBE/Au/SDD+2*f*/M/ECP28MWB level of theory. The simulated anion photoelectron spectra company with structures of the several candidate lowest-energy isomers (approx. 0.3 eV) of MAu_16_^−^ (M = Mo and Tc) are shown in figures 4 and 5. The spin multiplicity (SM), symmetry type (Sym), relative energy (Δ*E*), binding energy (BE) per atom, doping energy (DE) per atom, HOMO–LUMO energy gap (*E*_gap_), and calculated first ADE and VDE for the top five of M@Au_16_^−^ (M = Mo and Tc) are shown in figures 2 and 3 and also given in table 2.

**Table 2.** Structural and energetic characteristics for the five low-lying isomers of doped cluster anions M@Au_16_^−^ (M = Mo and Tc).*^a^* All energies are given in eV.

cluster	structure	SM	Sym	*ΔE*	BE	DE	*E*_gap_	ADE	VDE
Mo@Au_16_^−^	a	2	*C*_2v_	0.00	2.48	0.29	0.40	3.24	3.29
	b	2	*C*_1_	0.43	2.45	0.27	0.32	2.66	3.22
	c	2	*C*_1_	0.41	2.45	0.27	0.32	3.05	3.16
	d	2	*C*_1_	0.52	2.45	0.26	0.33	3.08	3.19
	e	2	*C*_1_	0.66	2.44	0.25	0.34	3.00	3.11
Tc@Au_16_^−^	a	1	*C*_2v_	0.10	2.46	0.28	0.45	3.10	3.18
	b	5	*C*_1_	0.00	2.46	0.29	0.51	3.31	3.43
	c	5	*C*_1_	0.18	2.45	0.28	0.24	2.85	2.96
	d	7	*C*_s_	0.09	2.46	0.29	0.16	2.81	2.91
	f	7	*C*_1_	0.22	2.45	0.28	0.13	2.94	3.02

*^a^*Shown are the spin multiplicity (SM), symmetry type (Sym), relative energy (Δ*E*), binding energy (BE) per atom, doping energy (DE) per atom, HOMO–LUMO energy gap (*E*_gap_), and calculated first ADE/VDE.

### 3.1. Geometric structure and stability

For the neutral and anionic host cluster Au_16_, we present two important types of structures: a perfect cage *D*_2d_ structure A and a high symmetric planar *C*_2v_ structure B. It is somewhat surprising that anionic structure A is 0.042 eV above the planar structure B at PBEPBE/SDD+2*f* level of theory; however, calculated first ADE and VDE of structure A are in accord with experimental PES data [18,27] and better than those of structure B (ADE/VDE, theoretical: 3.89/4.02 eV for A, 3.75/3.78 eV for B; experimental: 3.99 ± 0.03/4.03 ± 0.03 eV, table 1). Our recent study [23] found that the global minimum structure A of Au_16_^−^ cluster with *C*_2_ symmetry is (about 0.10 eV) more stable than the planar structure B at PBEPBE/LANL2DZ level. To clearly compare simulated PES with the experimental PES, we present RMSD for the structures A and B, the smallest RMSD value (0.06 eV) for isomer A implies the closest match to experimental PES (table 1). In other words, the simulated PES of the *D*_2d_ isomer A obviously agrees better than that of *C*_2v_ isomer B with the experimental spectrum (figure 1). These results lead us to conclude that the structure A should be a reasonable isomer in the Au_16_^−^ gas cluster beam. For neutral Au_16_, isomer A is less stable than isomer B by 0.639, 0.352, 0.181 and 0.155 eV at four functionals B3LYP, BP86, PBE and PW91, respectively, indicating that planar isomer is the most possible structure. In order to compare the degree of structural change of structure A under different point group symmetry, we calculate the average bond length. The results show that the average bond length of anionic Au_16_ is 2.85, 2.81, 2.80, 2.80, 2.78 Å for B3LYP, BP86, PBE, PW91, TPSS functional, respectively, and for neutral Au_16_, the calculated average length using the five functional is 2.86, 2.81, 2.81, 2.81, 2.79 Å. As can be seen, whether for neutral cluster or anionic Au_16_ cluster, the calculated values using BP86, PBE, PW91 and TPSS functionals are close to each other (about 2.80 Å for Au_16_ and 2.81 Å for Au_16_^−^), indicating that structure distortion of these isomers calculated using different functionals is not obvious. The result of B3LYP functional is a bit larger than those of other functionals. B3LYP functional is not suitable for the study of gold clusters, which has been confirmed by the study in the pure golden clusters Au*_n_*^−^ (*n* = 16–18) [27].

One of the most important questions that we want to address here is to discover the lowest-energy structures of the MAu_16_^−^ clusters. We have obtained many isomers and determined the lowest-energy structures for MAu_16_^−^ (M = Mo and Tc) using the computation scheme SK-DFT described in §2. The ground-state structures and some low-lying metastable isomers of MAu_16_ cluster anions accompanied with relative energies are given in figures 2 and 3, where a blue sphere refers to the impurity atom M. All the low-lying isomers are formed in three different configurations, i.e. endohedral cages (isomers a–f), plane structures (isomers g–i) and exohedral derivatives (isomers j–k). For MoAu_16_^−^, all the 11 lowest-energy isomers are found to prefer the lowest spin state. However, the stability of isomers of TcAu_16_^−^ cluster is lower along with the increase of the spin multiplicities. Specifically, multiplicities vary substantially in some cases (SM = 2 for MoAu_16_^−^-d, but 7 for TcAu_16_^−^-d, similar situations occur in structures e–j). This may be attributed to the fact that the valence electron configuration of Mo is 4d^5^5s^1^, the unpaired electron in the 5s orbital is easily hybridized with 4d ones to form 4d–5s hybridization, thus reducing spin multiplicity. For TcAu_16_^−^, the valence electron configuration of Tc is 4d^5^5s^2^. 4d orbit is just a half-filled shell and two electrons fill the 5s orbit; because of this the electron structure is not easy to break, thereby making the isomer d of TcAu_16_^−^ high spin. Our SK-DFT calculations show that the six candidate lowest-energy isomers for MAu_16_^q^ are almost all low-symmetry ‘endohedral cages' with a dopant atom trapping inside the cage. Exohedral and plane structures for MAu_16_^−^ are obviously less stable than endohedral structures. As shown in figures 2 and 3, the top-six lowest-lying isomers of MoAu_16_^−^ resemble closely the shape of TcAu_16_^−^ clusters, although the order of total energies is completely dissimilar to each other. For the MoAu_16_^−^ cluster, an endohedral structure (a) with relatively high symmetry (*C*_2v_) was obtained, which can be derived from the previously discovered M@Au_12_^−^ (M = W and Mo) icosahedral structure [65] with high symmetry and significant stability by capping four additional Au atoms on two triangular broadside faces. Other five endohedral isomers from MoAu_16_^−^ (b) to MoAu_16_^−^ (f) with low symmetry were less stable than the global minimum structures by 0.41–0.87 eV at high-level PBEPBE/Au/SDD+2*f*/M/ECP28MWB. Considering the large relative energies for such isomers, we can rule out the possibility that these higher energy isomers become the ground-state structure. As for TcAu_16_^−^ cluster, the global minimum structure (b) has a quintet state with low symmetry C_1_. However, five structures (isomers a, b, c, d, f) are found to be close to each other in energy (approximately 0.3 eV, figure 3). Especially, isomers a and b are found to be degenerated in energy at PBEPBE/LANL2DZ level, while isomers a and d are found to be nearly degenerated in energy at PBEPBE/Au/SDD+2*f*/M/ECP28 MWB. Keeping in mind the inherent accuracy of the DFT, we could not exclude the probability of four isomers by DFT calculations. It is necessary to compare their simulated photoelectron spectra with the experimental photoelectron spectra. Unfortunately, without the experimental photoelectron spectra for TcAu_16_^−^ cluster, we only provide theoretical photoelectron spectra in the present work. In order to compare the various local minimum structures with other 3d or 4d transition metal atom doping, we also present the first three planar shape isomers and two exohedral structures. The first three two-dimensional (2D) planar local minimum structures possess high symmetry by SK-DFT at PBEPBE/Au/SDD+2*f*/M/ECP28MWB level. As seen from figure 2, the two 2D structures g and i have a relatively high symmetry *C*_2v_, which are less stable than the ground-state structure a by 1.81 and 4.39 eV for MoAu_16_^−^. The similar situation occurred for TcAu_16_^−^, where 2D isomers g and i present the same symmetry *C*_2v_ and possess the higher energy than lowest-energy structure a by 1.52 and 2.43 eV. The structures with exohedral doping are all higher-lying isomers, this is in accord with the results of other 3d and 4d transition metal atoms (e.g. Sc, Ti, V, Cr, Mn, Fe, Co, Ni, Cu, Zn, Y, Zr and Nb) doping in golden cage cluster Au_16_^−^ [18–20,22–24]. It is thoroughly different from the doping of a group IV atom (Si, Ge and Sn) into the Au_16_^−^ cage [30], forming the exohedral gold cluster M@Au_16_^−^. Furthermore, the exohedral doping ground-state structure also occurred for C-, K- and Ag-doped golden clusters, as shown by Fa & Yang [16,32].

Table 2 shows the binding energy, doping energy and HOMO–LUMO gap of Mo@Au_16_^−^ and Tc@Au_16_^−^ clusters. For the Mo@Au_16_^−^ cluster, structure a has the largest binding energy of 2.48 eV among all the isomers. The binding energy of isomers b, c, d and e is about 2.44 to 2.45 eV, which is smaller than that of the isomer a. For the doping energy, it is defined as the energy sum of the Au_16_^−^ and the M atom minus the total energy of the MAu_16_^−^, as given in table 2, the doping energy of isomer a is larger than that of other ones, indicating that isomer a has the highest stability among all the isomers. As for the HOMO–LUMO gap, it can be seen from table 2 that isomer a has obviously higher HOMO–LUMO gap relative to isomers b, c, d and e. The HOMO–LUMO gap of isomer a is 0.40 eV, and the values of other four isomers are approximately 0.33 eV. For Tc@Au_16_^−^ cluster, the binding energy and the doping energy of the lowest-energy isomers are close to each other. The values of about 2.46 and 0.28 eV are for binding energy and doping energy, respectively. For the HOMO–LUMO gap, the result of isomer b is significantly higher than those of isomers a, c, d and f, suggesting that the isomer b is the most chemically stable. The lowest-energy structures Mo@Au_16_^−^-a and Tc@Au_16_^−^-b stand out in the stability, as measured by their high binding energy per atom (greater than 2.46 eV), doping energy (0.29 eV) as well as a large HOMO–LUMO gap (greater than 0.40 eV).

### 3.2. Photoelectron spectroscopy and detachment energy

The first ADE is determined by calculating the energy difference between the optimized geometry of the anionic cluster and the optimized neutral cluster as the initial point at the anion geometry. Single-point energies of the neutral clusters are also computed based on the same optimized anion geometry. The difference in the energy of the anion and neutral cluster gives the first VDE. We examined 11 low-lying isomers by energies from the PBEPBE/Au/SDD+2*f*/M/ECP28MWB level, including six endohedral cages (isomers a–f), three plane structures (isomers g–i) and two exohedral doping (isomers j–k), but only one candidate (isomer a) for MoAu_16_^−^ and five candidates (isomers a–d) for TcAu_16_^−^ approximately 0.3 eV. In order to assist the future comparison with further experiments, we have drawn the simulated anion photoelectron spectra company with structures of the several candidate lowest-energy isomers (approximately 0.3 eV) of M@Au_16_^−^ (M = Mo and Tc) in figures 4 and 5. Those isomers with the values approximately 0.3 eV from the lowest-energy isomer are all regarded as candidates for the low-lying isomers. The calculated first ADE and VDE for the top five of MAu_16_^−^ (M = Mo and Tc) are presented in table 2. The first VDE of each cluster anion corresponds to the first peak maximum of each spectrum in figures 4 and 5. The ADE/VDE of the top five of M@Au_16_^−^ are 3.24/3.29 (a), 2.66/3.22 (b), 3.05/3.16 (c), 3.08/3.19 (d), 3.00/3.11 eV (e) for M = Mo, and 3.10/3.18 (a), 3.31/3.43 (b), 2.85/2.96 (c), 2.81/2.91 (d), 2.94/3.02 eV (f) for M = Tc, which are smaller than those of Au_16_^−^ cage (theoretical: 3.89/4.02 eV, experimental: 3.99 ± 0.03/4.03 ± 0.03 eV [18,27], table 1). The energy difference between ADE and VDE is very little, e.g. the difference in energy is only 0.05 eV for Mo@Au_16_^−^-(a) and 0.08, 0.12, 0.11, 0.10, 0.08 eV for Tc@Au_16_^−^-(a)(b)(c)(d)(f), respectively. This is due to the negligible geometry changes between the anionic and neutral ground-state structures.

As illustrated in figures 4 and 5, the simulated spectra of the several low-lying isomers in both MoAu_16_^−^ and TcAu_16_^−^ are a lot different from those of the pure Au_16_^−^ cage, reflecting their geometric structure with a large change, meanwhile, with a larger energy gap (X–A) than the pure cluster. Remarkably, the simulated photoelectron spectra of MAu_16_^−^ (figures 4 and 5) consist of rather congested PES features with large intensity variations. However, the PES spectrum of Au_16_^−^ is rather simple relative to its neighbouring sizes, does not exhibit a large energy gap like other even-sized gold clusters [66], because the tetrahedral Au_16_ cage is an open shell with two unpaired electrons and two extra electrons are needed to make a closed-shell 18-electron Au_16_^2−^ cage. The simulated PES spectrum of *C*_2v_ Mo@Au_16_^−^ (a) is predicted to have a low first vertical detachment energy at 3.29 eV, which is even lower than that of *D*_2d_ Au_16_^−^ (experimental first VDE: 4.03 ± 0.03 eV). The neutral Mo@Au_16_ cluster with 22 valence electrons possesses a closed-shell configuration, as evident from the theoretical PES spectrum of *C*_2v_ Mo@Au_16_^−^ (a) which exhibits a sizable HOMO–LUMO gap of approximately 0.40 eV, and approximately 0.14 eV larger than the experimental energy gap of Au_16_^−^. The lowest-energy structure Tc@Au_16_^−^ (b) and the others four isomers Tc@Au_16_^−^ (a), (c), (d), (f) are separated by only 0.10, 0.18, 0.09 and 0.22 eV, respectively, at PBEPBE/Au/SDD+2*f*/M/ECP28MWB level. Considering the uncertainty of the DFT energies for such systems, we could not conclude which isomer structure should be assigned as the global minimum for TcAu_16_^−^ cluster. Unfortunately, no gas-phase experimental photoelectron spectra literature can be acquired for TcAu_16_^−^ cluster. The calculated ADE/VDE values of the top-five low-lying isomers Tc@Au_16_^−^ (a)–(d) and (f) are also less than the experimental values of pure clusters Au_16_^−^. The predicted PES spectra of the top-five lowest-energy structures of Tc@Au_16_^−^ are somewhat similar, each theoretical PES with no less than 12 relatively sharp peaks and comparatively broader HOMO–LUMO gap (X–A gap). The spectrum of lowest-energy structure Tc@Au_16_^−^ (b) presents four well-resolved peaks in the binding energy range 3–4 eV followed by the less congested spectral features above 4 eV. The PES spectra of the doped cluster of Tc@Au_16_^−^ with the inside Tc atom present different features from those of the parent hollow cage, suggesting that its geometric and electronic structures alter remarkably due to the addition of Tc atom. Our SK-DFT results also support this point of view, indeed, there are significant differences in their geometric structure compared with that of the pure truncated tetrahedron cage. We cannot rule out the possibility that these low-lying isomers of Tc@Au_16_^−^ exist in gas-phase cluster beam by simulated PES spectra and small relative energies. However, we can predict isomer Tc@Au_16_^−^ (b) to be the major species and other isomers Tc@Au_16_^−^ (a), (c), (d), (f) to be the minor ones contributing to the photoelectron spectrum of TcAu_16_^−^ cluster in the future experiments. It will be very interesting to measure the PES for validating our predicted results on the Mo- and Tc-doped Au_16_ clusters. The predicted photoelectron spectra should aid in the forthcoming experimental characterization of MAu_16_^−^ (M = Mo and Tc) clusters.

## 4. Conclusion

We present a combined Saunders ‘Kick' global search technique and density-functional theory study of anionic and neutral doped golden cage clusters MAu_16_ (M = Mo and Tc). The global minimum search revealed that the endohedral cages represent the global minimum structure for the doped gold clusters MAu_16_^q^. Three different structures including endohedral cages, plane and exohedral structures are obtained using the SK-DFT method. The structures of Mo- and Tc-doped endohedral cages exhibit larger distortion from the bare gold cluster cage. Based on small relative energies, we can predict that structure Tc@Au_16_^−^ (b) to be the major species and other isomers Tc@Au_16_^−^(a), (c), (d), (f) to be the minor ones contributing to the photoelectron spectrum of TcAu_16_^−^ in the future experiments. Moreover, theoretical PES spectra of the leading candidate clusters also have been predicted. It will be very meaningful if the leading candidate clusters presented here could be detected by the future PES experiment for validating the existence of the Mo- and Tc-doped Au_16_ clusters. This structural information on low-symmetry endohedral cage clusters could be considered as the building blocks for cluster-assembled materials.

## Supplementary Material

Cartesian coordinates of low-lying isomers of Au16 and MAu16 clusters

## Data accessibility

The datasets supporting this article have been uploaded as part of the electronic supplementary Material.
