# Supplementary material for: Stabilization of golden cages by encapsulation of a single transition metal atom
Source: R Soc Open Sci. 2018 Jan 3;5(1):171019. doi: 10.1098/rsos.171019 (PMC5792890; doi:10.1098/rsos.171019)
Supplement: Cartesian coordinates of low-lying isomers of Au16 and MAu16 clusters [file rsos171019supp1.doc]

Stabilization of golden cages by encapsulation of a single transition metal atom

Hui-Fang Li and Huai-Qian Wang*

*College of Engineering, Huaqiao University, Quanzhou, 362021, China.*

^*^E-mail: [hqwang@hqu.edu.cn](mailto:hqwang@hqu.edu.cn)

**Table of Contents**

**Table S1.** Cartesian coordinates for stable isomers of Au_16_^–^ cluster obtained at five different methods.

**Table S2.** Cartesian coordinates for stable isomers of Au_16_ cluster obtained at five different methods.

**Table S3.** Cartesian coordinates for stable isomers of MAu_16_^–^ (M=Mo and Tc) clusters obtained at PBEPBE/Au/SDD+2f/M/ECP28MWB level of theory.

**Table S1.** Cartesian coordinates for stable isomers of Au_16_^–^ cluster obtained at five different methods.

**B3LYP-A**

Au -1.51384300 -1.66303400 1.26025000

Au -1.51754100 1.66268200 -1.25912200

Au -3.02740600 0.82677600 1.09568600

Au 1.51598400 1.26041300 1.66155700

Au -1.02522600 2.75722800 1.28381500

Au -1.02173100 0.48868200 3.00218400

Au -3.02829400 -0.82955500 -1.09158000

Au 1.02691900 -1.28255400 2.75706700

Au 3.02694900 1.09512700 -0.82953900

Au 1.02267200 1.28366600 -2.75831800

Au 3.02888400 -1.09260300 0.82643600

Au 1.51537900 -1.25936900 -1.66377700

Au 1.02508000 -3.00163600 0.48887400

Au -1.02430100 -2.75851800 -1.28261500

Au -1.02544100 -0.48969500 -3.00112200

Au 1.02191600 3.00238900 -0.48979700

**B3LYP-B**

Au 0.00000000 2.78224500 3.87302000

Au 0.00000000 4.22168000 1.54289100

Au 0.00000000 5.53789000 -0.85405700

Au 0.00000000 0.00000000 3.92788800

Au 0.00000000 1.38566400 1.48571800

Au 0.00000000 2.76875200 -0.89781300

Au 0.00000000 4.12978800 -3.27497900

Au 0.00000000 -2.78224500 3.87302000

Au 0.00000000 -1.38566400 1.48571800

Au 0.00000000 0.00000000 -0.92815900

Au 0.00000000 1.36503400 -3.37464400

Au 0.00000000 -4.22168000 1.54289100

Au 0.00000000 -2.76875200 -0.89781300

Au 0.00000000 -1.36503400 -3.37464400

Au 0.00000000 -5.53789000 -0.85405700

Au 0.00000000 -4.12978800 -3.27497900

**BP86-A**

Au -1.46056200 0.56715300 -1.94694500

Au -1.46607400 -0.56720800 1.94391200

Au -2.97288700 -1.29963000 -0.38199200

Au 1.46367700 -1.94522700 -0.56511600

Au -0.99985600 -2.95916200 0.59403200

Au -0.99763700 -2.17504700 -2.09203800

Au -2.97387500 1.29990200 0.37458500

Au 1.00305900 -0.59567400 -2.95664300

Au 2.97057700 -0.37851400 1.30243700

Au 0.99567400 0.59527500 2.95934000

Au 2.97400200 0.37786800 -1.29529000

Au 1.46292100 1.94555100 0.56863200

Au 1.00385700 2.09114700 -2.17364600

Au -0.99809000 2.95945200 -0.59677200

Au -1.00278700 2.17549500 2.08990400

Au 0.99800100 -2.09137900 2.17559900

**BP86-B**

Au 0.00000000 2.74102500 3.82836800

Au 0.00000000 4.15633800 1.52412500

Au 0.00000000 5.46023800 -0.84007800

Au 0.00000000 0.00000000 3.87565400

Au 0.00000000 1.36912600 1.46498300

Au 0.00000000 2.72520600 -0.88543600

Au 0.00000000 4.07028100 -3.23240100

Au 0.00000000 -2.74102500 3.82836800

Au 0.00000000 -1.36912600 1.46498300

Au 0.00000000 0.00000000 -0.92366500

Au 0.00000000 1.34507400 -3.33555400

Au 0.00000000 -4.15633800 1.52412500

Au 0.00000000 -2.72520600 -0.88543600

Au 0.00000000 -1.34507400 -3.33555400

Au 0.00000000 -5.46023800 -0.84007800

Au 0.00000000 -4.07028100 -3.23240100

**PBE-A**

Au 0.86947400 1.80458500 -1.44731200

Au -0.86947400 -1.80458500 -1.44731200

Au 1.22034500 -0.58758400 -2.97172300

Au 1.80459100 -0.86943900 1.44727600

Au 1.80459100 -2.42118100 -0.99847100

Au 3.01816600 0.09811800 -0.99809800

Au -1.22034500 0.58758400 -2.97172300

Au 2.42155500 1.80477800 0.99829200

Au -0.58772400 -1.22067700 2.97213700

Au -2.42155500 -1.80477800 0.99829200

Au 0.58772400 1.22067700 2.97213700

Au -1.80459100 0.86943900 1.44727600

Au -0.09819000 3.01851900 0.99789800

Au -1.80459100 2.42118100 -0.99847100

Au -3.01816600 -0.09811800 -0.99809800

Au 0.09819000 -3.01851900 0.99789800

**PBE-B**

Au 0.00000000 2.73959900 3.82620700

Au 0.00000000 4.15170900 1.52125400

Au 0.00000000 5.45923000 -0.84042800

Au 0.00000000 0.00000000 3.87135400

Au 0.00000000 1.36829400 1.46439800

Au 0.00000000 2.72533900 -0.88516200

Au 0.00000000 4.07031800 -3.23124900

Au 0.00000000 -2.73959900 3.82620700

Au 0.00000000 -1.36829400 1.46439800

Au 0.00000000 0.00000000 -0.92168500

Au 0.00000000 1.34522000 -3.32985500

Au 0.00000000 -4.15170900 1.52125400

Au 0.00000000 -2.72533900 -0.88516200

Au 0.00000000 -1.34522000 -3.32985500

Au 0.00000000 -5.45923000 -0.84042800

Au 0.00000000 -4.07031800 -3.23124900

**PW91-A**

Au -1.45601700 1.70122600 -1.05542600

Au -1.44319300 -1.70808300 1.06105300

Au -2.96988400 -0.72085400 -1.14443100

Au 1.44853600 -1.05486400 -1.70755700

Au -0.99646000 -2.60031600 -1.53246600

Au -1.00462500 -0.22378700 -3.00700000

Au -2.96858300 0.70726700 1.15578300

Au 0.99025800 1.53666200 -2.59946200

Au 2.97408600 -1.14339200 0.70839800

Au 1.00865300 -1.53179100 2.59616200

Au 2.96512200 1.15789400 -0.71982600

Au 1.44997000 1.06156600 1.70192500

Au 0.99110500 3.01176600 -0.22321300

Au -1.00290300 2.59504100 1.53582700

Au -0.99359900 0.21936100 3.01101700

Au 1.00753400 -3.00769700 0.21921600

**PW91-B**

Au 0.00000000 2.73728900 3.82411300

Au 0.00000000 4.14991600 1.52126200

Au 0.00000000 5.45491900 -0.84001200

Au 0.00000000 0.00000000 3.87023300

Au 0.00000000 1.36734500 1.46373100

Au 0.00000000 2.72290400 -0.88448300

Au 0.00000000 4.06677700 -3.22920500

Au 0.00000000 -2.73728900 3.82411300

Au 0.00000000 -1.36734500 1.46373100

Au 0.00000000 0.00000000 -0.92187900

Au 0.00000000 1.34412600 -3.32958300

Au 0.00000000 -4.14991600 1.52126200

Au 0.00000000 -2.72290400 -0.88448300

Au 0.00000000 -1.34412600 -3.32958300

Au 0.00000000 -5.45491900 -0.84001200

Au 0.00000000 -4.06677700 -3.22920500

**TPSS-A**

Au -1.38373700 1.52005900 -1.15154300

Au -1.37483200 -1.52942900 1.14896900

Au -2.94708700 -0.82407100 -1.08185200

Au 1.38470500 -1.14549500 -1.52263800

Au -0.98086900 -2.72570600 -1.29965300

Au -0.98630100 -0.50232200 -2.97418800

Au -2.95562300 0.80361900 1.07574100

Au 0.98951100 1.30175500 -2.72073500

Au 2.95479700 -1.06886400 0.81633300

Au 0.99393200 -1.29553200 2.72322200

Au 2.94827500 1.08916900 -0.81133100

Au 1.37362400 1.15501900 1.52584000

Au 0.98145900 2.97783700 -0.49817900

Au -1.00315500 2.71950800 1.29754800

Au -0.99647800 0.49546200 2.97309900

Au 1.00178000 -2.97100800 0.49936700

**TPSS-B**

Au 0.00000000 2.72253700 3.80355400

Au 0.00000000 4.12293600 1.50860400

Au 0.00000000 5.42621100 -0.83997500

Au 0.00000000 0.00000000 3.84705700

Au 0.00000000 1.36058600 1.45730800

Au 0.00000000 2.70975000 -0.88021200

Au 0.00000000 4.04596900 -3.21276100

Au 0.00000000 -2.72253700 3.80355400

Au 0.00000000 -1.36058600 1.45730800

Au 0.00000000 0.00000000 -0.91394300

Au 0.00000000 1.33756500 -3.30307500

Au 0.00000000 -4.12293600 1.50860400

Au 0.00000000 -2.70975000 -0.88021200

Au 0.00000000 -1.33756500 -3.30307500

Au 0.00000000 -5.42621100 -0.83997500

Au 0.00000000 -4.04596900 -3.21276100

**Table S2.** Cartesian coordinates for stable isomers of Au_16_ cluster obtained at five different methods.

**B3LYP-A**

Au -1.47185700 1.38774600 1.38725400

Au -1.47507600 -1.38775000 -1.38605000

Au -3.08719400 0.98974700 -0.98617900

Au 1.47276100 1.38565600 -1.38841100

Au -1.08281000 0.83381900 -2.93244600

Au -1.07969600 2.93369800 -0.83506000

Au -3.08646900 -0.98778700 0.99021600

Au 1.08281100 2.93445700 0.83146200

Au 3.08597800 -0.98924500 -0.99070800

Au 1.08044600 -2.93463700 -0.83321400

Au 3.08807300 0.98804500 0.98652000

Au 1.47443700 -1.38709000 1.38728000

Au 1.08151300 0.83646600 2.93165300

Au -1.07927000 -0.83315300 2.93426600

Au -1.08043500 -2.93321600 0.83621000

Au 1.07678700 -0.83675500 -2.93279300

**B3LYP-B**

Au 0.00000000 2.79773100 3.81010500

Au 0.00000000 4.23916300 1.52816200

Au 0.00000000 5.56882400 -0.83674400

Au 0.00000000 0.00000000 3.95081800

Au 0.00000000 1.35643000 1.48628900

Au 0.00000000 2.79012200 -0.88006500

Au 0.00000000 4.09236000 -3.24368200

Au 0.00000000 -2.79773100 3.81010500

Au 0.00000000 -1.35643000 1.48628900

Au 0.00000000 0.00000000 -0.94449900

Au 0.00000000 1.35444400 -3.36722400

Au 0.00000000 -4.23916300 1.52816200

Au 0.00000000 -2.79012200 -0.88006500

Au 0.00000000 -1.35444400 -3.36722400

Au 0.00000000 -5.56882400 -0.83674400

Au 0.00000000 -4.09236000 -3.24368200

**BP86-A**

Au 1.39929700 1.73844300 -0.73925100

Au 1.39430800 -1.74395200 0.73918200

Au 3.03312500 0.53012500 1.26455000

Au -1.39529200 0.74112100 1.74106200

Au 1.04509000 -0.29259600 3.00637800

Au 1.05630100 2.36448400 1.87980400

Au 3.03154500 -0.53844500 -1.26482300

Au -1.03938700 3.00850100 0.29073700

Au -3.03553500 -1.26000400 0.53406900

Au -1.05060600 -3.00503400 -0.29181900

Au -3.03011700 1.27068600 -0.53407000

Au -1.39856600 -0.73747700 -1.74119200

Au -1.04876100 1.88171200 -2.36547700

Au 1.04567000 0.28919600 -3.00593000

Au 1.04890100 -2.36770800 -1.87941300

Au -1.05597400 -1.87905100 2.36619300

**BP86-B**

Au 0.00000000 2.75179300 3.77841600

Au 0.00000000 4.16610100 1.51160300

Au 0.00000000 5.48696700 -0.82095000

Au 0.00000000 0.00000000 3.88708700

Au 0.00000000 1.34441900 1.46091200

Au 0.00000000 2.73998700 -0.86931200

Au 0.00000000 4.04050600 -3.20338600

Au 0.00000000 -2.75179300 3.77841600

Au 0.00000000 -1.34441900 1.46091200

Au 0.00000000 0.00000000 -0.94006600

Au 0.00000000 1.33525400 -3.33079400

Au 0.00000000 -4.16610100 1.51160300

Au 0.00000000 -2.73998700 -0.86931200

Au 0.00000000 -1.33525400 -3.33079400

Au 0.00000000 -5.48696700 -0.82095000

Au 0.00000000 -4.04050600 -3.20338600

**PBE-A**

Au -1.35872400 -0.49262800 1.77546100

Au -1.35782100 0.49237700 -1.77649300

Au -3.03247900 1.32719200 0.36749700

Au 1.35796200 1.77599700 0.49297200

Au -1.04891800 2.95246400 -0.68048400

Au -1.04923600 2.17955900 2.10408700

Au -3.03237500 -1.32717000 -0.36903100

Au 1.04835200 0.68003600 2.95215300

Au 3.03230300 0.36829600 -1.32566900

Au 1.05026500 -0.67996800 -2.95151300

Au 3.03149600 -0.36788000 1.32741500

Au 1.35871000 -1.77649200 -0.49213400

Au 1.04823800 -2.10435200 2.17954200

Au -1.04935500 -2.95216700 0.68017100

Au -1.04787700 -2.17982800 -2.10476500

Au 1.04945900 2.10456500 -2.17920900

**PBE-B**

Au 0.00000000 2.75168500 3.77674700

Au 0.00000000 4.16171000 1.50826900

Au 0.00000000 5.48534500 -0.82195600

Au 0.00000000 0.00000000 3.88229000

Au 0.00000000 1.34415600 1.46070600

Au 0.00000000 2.73949500 -0.86956100

Au 0.00000000 4.04021500 -3.20263400

Au 0.00000000 -2.75168500 3.77674700

Au 0.00000000 -1.34415600 1.46070600

Au 0.00000000 0.00000000 -0.93687500

Au 0.00000000 1.33506900 -3.32427900

Au 0.00000000 -4.16171000 1.50826900

Au 0.00000000 -2.73949500 -0.86956100

Au 0.00000000 -1.33506900 -3.32427900

Au 0.00000000 -5.48534500 -0.82195600

Au 0.00000000 -4.04021500 -3.20263400

**PW91-A**

Au -1.36817900 0.07265700 1.85045200

Au -1.36617900 -0.07106600 -1.85205000

Au -3.02956300 1.37514700 -0.05575100

Au 1.36798600 1.85114900 -0.07128000

Au -1.04294300 2.60086000 -1.54374100

Au -1.04863500 2.71385200 1.33846200

Au -3.03091100 -1.37253800 0.05263000

Au 1.04436700 1.54324900 2.60093700

Au 3.03083400 -0.05586700 -1.37203000

Au 1.04615000 -1.54411300 -2.59995200

Au 3.02928700 0.05298700 1.37537900

Au 1.36578200 -1.85204400 0.07263300

Au 1.04706900 -1.33996800 2.71419800

Au -1.04725500 -2.60027200 1.54259000

Au -1.04952200 -2.71333500 -1.33974700

Au 1.05171000 1.33930000 -2.71273000

**PW91-B**

Au 0.00000000 2.74964700 3.77527000

Au 0.00000000 4.15955300 1.50830800

Au 0.00000000 5.48060900 -0.82115500

Au 0.00000000 0.00000000 3.88173000

Au 0.00000000 1.34344600 1.45951300

Au 0.00000000 2.73771200 -0.86882900

Au 0.00000000 4.03642300 -3.20065100

Au 0.00000000 -2.74964700 3.77527000

Au 0.00000000 -1.34344600 1.45951300

Au 0.00000000 0.00000000 -0.93753800

Au 0.00000000 1.33412200 -3.32455100

Au 0.00000000 -4.15955300 1.50830800

Au 0.00000000 -2.73771200 -0.86882900

Au 0.00000000 -1.33412200 -3.32455100

Au 0.00000000 -5.48060900 -0.82115500

Au 0.00000000 -4.03642300 -3.20065100

**TPSS-A**

Au 1.17627900 -1.33635200 -0.90385700

Au 1.17490400 1.33240200 0.90972800

Au 3.03001400 0.77118600 -1.13697900

Au -1.16845300 0.90804000 -1.33642300

Au 1.06753200 2.70697700 -1.39327800

Au 1.06400700 0.30140500 -3.02745400

Au 3.01964300 -0.78186200 1.15542300

Au -1.05213100 -1.39456700 -2.71122000

Au -3.02508200 1.15171900 0.76741000

Au -1.06335900 1.39818100 2.70518300

Au -3.02456400 -1.14083700 -0.78581600

Au -1.18315800 -0.90521900 1.33157400

Au -1.05841100 -3.02828300 -0.30612100

Au 1.04847800 -2.70983600 1.39924500

Au 1.04379600 -0.30512200 3.03262100

Au -1.04949500 3.03216700 0.29996500

**TPSS-B**

Au 0.00000000 2.73468800 3.75773200

Au 0.00000000 4.13039800 1.49709100

Au 0.00000000 5.45074800 -0.81924000

Au 0.00000000 0.00000000 3.85490200

Au 0.00000000 1.33814500 1.45252900

Au 0.00000000 2.72372900 -0.86667500

Au 0.00000000 4.01795400 -3.18654500

Au 0.00000000 -2.73468800 3.75773200

Au 0.00000000 -1.33814500 1.45252900

Au 0.00000000 0.00000000 -0.92781700

Au 0.00000000 1.32791300 -3.29843500

Au 0.00000000 -4.13039800 1.49709100

Au 0.00000000 -2.72372900 -0.86667500

Au 0.00000000 -1.32791300 -3.29843500

Au 0.00000000 -5.45074800 -0.81924000

Au 0.00000000 -4.01795400 -3.18654500

**Table S3.** Cartesian coordinates for stable isomers of MAu_16_^–^ (M=Mo and Tc) clusters obtained at PBEPBE/Au/SDD+2f/M/ECP28MWB level of theory.

***Endohedral Mo@Au_16_^–^ (a~f)***

**MoAu_16_^–^-*a***

Au 0.00000000 3.99561300 -2.72772600

Au 0.00000000 1.35815000 -3.21133900

Au 0.00000000 2.40938000 -0.52289200

Au -2.31133500 -1.47460300 0.94267900

Au -1.47144900 0.00000000 3.27682300

Au 0.00000000 -2.36529400 2.38407800

Au 0.00000000 -2.40938000 -0.52289200

Au 1.62823300 0.00000000 -1.34012300

Au 2.31133500 -1.47460300 0.94267900

Au 0.00000000 -1.35815000 -3.21133900

Au 2.31133500 1.47460300 0.94267900

Au 1.47144900 0.00000000 3.27682300

Au -1.62823300 0.00000000 -1.34012300

Au 0.00000000 -3.99561300 -2.72772600

Au 0.00000000 2.36529400 2.38407800

Au -2.31133500 1.47460300 0.94267900

Mo 0.00000000 0.00000000 0.96237400

**MoAu_16_^–^-*b***

Au -3.60828300 0.65523800 -0.59730400

Au -2.39438900 -1.85888100 -1.46417500

Au -0.48380800 -0.51700300 2.68553300

Au -1.45631100 0.61274900 -2.59029700

Au 1.57987800 -0.72042600 0.82821800

Au 0.54524400 1.90979300 1.34500800

Au -2.91065600 -1.39908500 1.42298200

Au 2.37690200 3.51652000 -0.17868000

Au 3.50849200 1.10310200 0.05978700

Au 2.01380700 -3.18752000 -0.40780700

Au 1.04175200 1.35812200 -1.40959500

Au 0.47171500 -1.36567100 -1.89505500

Au -1.43125300 2.64531900 -0.58605900

Au -2.33092900 1.47933900 1.89462800

Au -0.54621400 -2.71549800 0.56735200

Au 4.17065800 -1.50181200 0.30515700

Mo -1.02813500 -0.02687200 0.03819600

**MoAu_16_^–^-*c***

Au -0.68324400 1.47297900 1.64971800

Au 1.56069600 -0.00023000 2.65875800

Au -0.06764300 -2.33614000 -1.02665500

Au -0.68228000 -1.47439100 1.64967600

Au 1.92073900 -2.36948800 1.13704200

Au 2.69459500 -1.76045800 -1.50757900

Au -2.78902900 -2.20574400 -0.16116200

Au -2.78921800 2.20536900 -0.16036700

Au -4.35768400 0.00014700 -0.85735400

Au 0.86034400 0.00005600 -2.74219000

Au -1.58815600 -0.00025000 -1.39307300

Au 3.76204300 0.00087600 0.55942500

Au 2.69253300 1.76215800 -1.50700600

Au -0.06895300 2.33667300 -1.02586300

Au -2.99968900 -0.00109100 1.61509200

Au 1.91897200 2.36961200 1.13804400

Mo 1.15861600 -0.00014600 -0.04985700

**MoAu_16_^–^-*d***

Au 0.49344400 1.65243200 2.32512700

Au 2.95369400 0.44905600 -1.41278800

Au 0.81170700 -1.25106200 2.32027100

Au 2.95340100 0.44790600 1.41351300

Au 4.80880600 -1.15910300 0.00037800

Au 1.68236300 2.71752100 0.00036000

Au 2.20567200 -2.12581500 -0.00027500

Au -3.21728500 -1.79673500 -0.00074600

Au -3.59053400 1.19784400 0.00003500

Au -0.58452200 -2.35752100 -0.00091900

Au -1.66682000 -0.06470900 -1.66656400

Au 0.81250200 -1.25033700 -2.32037200

Au 0.49386000 1.65258500 -2.32491700

Au -1.16487400 2.40587300 -0.00023600

Au -1.66722800 -0.06526700 1.66636900

Au -5.61169400 -0.55497500 0.00063200

Mo 0.54078900 0.19243600 0.00024900

**MoAu_16_^–^-*e***

Au 2.57599000 1.99516900 -1.21581100

Au -2.85931000 -2.33827100 -0.34973400

Au -0.14576800 2.27855900 -1.57014800

Au -4.33469700 -0.00073900 -0.22907900

Au -2.35110900 -0.00170200 1.73758500

Au 2.57380200 -1.99181000 -1.22578600

Au 0.30706900 -0.00119600 2.72276400

Au -1.61647200 0.00420800 -1.11035500

Au -2.86335600 2.34012200 -0.34224000

Au 2.20502400 1.80788300 1.67926300

Au 1.04648200 0.00163500 -2.74766100

Au -0.52694600 -2.11349900 1.15498100

Au -0.52381400 2.11106200 1.15209200

Au -0.14972700 -2.27524500 -1.56748500

Au 2.20171200 -1.81418100 1.67158900

Au 3.87079700 -0.00229200 0.26895700

Mo 1.11037100 0.00056000 -0.05441600

**MoAu_16_^–^-*f***

Au 0.08422600 0.17240600 -2.49340000

Au -1.15465800 -1.30389000 2.15252900

Au -1.94827700 -1.59258400 -0.69367800

Au -1.28658800 1.55250700 1.72486700

Au -4.54510300 -0.50213400 -1.24386300

Au -2.30027700 1.10774100 -1.13146400

Au 0.48486900 -2.43589100 -1.46445700

Au -0.14396100 2.64335600 -0.92634000

Au 1.35411800 2.35331900 1.48476600

Au 1.15570800 -2.46693100 1.29076100

Au 2.12765900 4.15378200 -0.58301900

Au 1.33135600 -0.05885500 2.71967600

Au 2.48780400 1.45812000 -0.96989500

Au 2.82769800 -1.11931300 -0.66351100

Au 2.78488500 -3.84700700 -0.53017100

Au -3.43412700 -0.10515500 1.24099000

Mo 0.32854300 -0.01781500 0.16215000

***Plane structures* *MoAu_16_*^–^ (*g*~*i*)**

**MoAu_16_^–^-*g***

Au 2.72857400 -4.13567200 -0.00000100

Au 4.12491500 -1.80883700 -0.00000100

Au 5.59097200 0.45569400 -0.00000200

Au -0.00038600 -4.11109000 0.00000000

Au 1.36122100 -1.71033400 0.00000200

Au 2.73854800 0.61100200 0.00000200

Au 4.23319700 2.81994000 -0.00000100

Au -2.72916900 -4.13513900 -0.00000100

Au -1.36147900 -1.71011800 0.00000200

Au 1.46869400 3.02399800 0.00000200

Au -4.12499200 -1.80835900 -0.00000100

Au -2.73844900 0.61124200 0.00000200

Au -1.46821600 3.02416600 0.00000200

Au -5.59093200 0.45611000 -0.00000200

Au -4.23294600 2.82043700 -0.00000100

Mo 0.00006100 0.65109200 0.00000300

Au 0.00041300 5.25081100 -0.00000200

**MoAu_16_^–^-*h***

Au -3.79859300 -3.12533600 0.00000000

Au -4.61961900 -0.53220000 0.00000000

Au -5.30619900 2.07870900 0.00000000

Au -1.16994500 -3.79351000 0.00000000

Au -1.91993500 -1.13572100 0.00000000

Au -2.65452800 1.48772900 0.00000000

Au -3.44624500 4.10381800 0.00000000

Au 1.47142900 -4.44801500 0.00000000

Au 0.75143500 -1.78430400 0.00000000

Au 0.00000000 0.83644500 0.00000000

Au -0.77674100 3.52466800 0.00000000

Au 3.38518900 -2.53459200 0.00000000

Au 2.72721000 0.18336600 0.00000000

Au 1.82826100 2.83043900 0.00000000

Au 5.40618300 -0.76573500 0.00000000

Au 4.48978600 2.35587600 0.00000000

Mo 6.83220500 1.35120500 0.00000000

**MoAu_16_^–^-*i***

Au 0.00000000 2.71029900 4.02799200

Au 0.00000000 4.16320300 1.71094700

Au 0.00000000 5.45527900 -0.65031600

Au 0.00000000 0.00000000 4.04154600

Au 0.00000000 1.38081400 1.63392300

Au 0.00000000 2.70771100 -0.71147300

Au 0.00000000 4.14268500 -3.04441200

Au 0.00000000 -2.71029900 4.02799200

Au 0.00000000 -1.38081400 1.63392300

Au 0.00000000 0.00000000 -0.76219200

Au 0.00000000 1.43919700 -3.19120700

Au 0.00000000 -4.16320300 1.71094700

Au 0.00000000 -2.70771100 -0.71147300

Au 0.00000000 -1.43919700 -3.19120700

Au 0.00000000 -5.45527900 -0.65031600

Au 0.00000000 -4.14268500 -3.04441200

Mo 0.00000000 0.00000000 -5.32358400

***Exohedral MoAu_16_^–^ (j~k)***

**MoAu_16_^–^-*j***

Au -0.35898800 -1.39894000 -1.88571600

Au -2.66972700 0.00179900 -1.07295100

Au -2.17172000 -1.46496000 1.31009600

Au 2.03547700 1.83821400 -0.30340200

Au 2.87185900 -0.00172300 1.65999900

Au 1.89782900 -0.00043600 -2.50319900

Au -2.16919200 1.46601700 1.31086300

Au -0.23088200 3.21767800 0.24092400

Au 4.07616200 -0.00226500 -0.87096800

Au -2.65202200 -2.80281000 -1.08523700

Au 2.03341300 -1.84052400 -0.30374000

Au 0.62619700 -1.50701600 2.24660700

Au -0.23533600 -3.21815800 0.24064200

Au -0.35657900 1.40053700 -1.88420100

Au 0.62714200 1.50650900 2.24687400

Au -2.64822100 2.80684300 -1.08324100

Mo -1.27041900 -0.00143800 3.26655200

**MoAu_16_^–^-*k***

Au 0.52520900 3.23630300 0.37190400

Au 0.13420200 -1.63671900 2.10289500

Au 1.99081600 -0.63320500 -1.79446300

Au -1.34811000 -3.16261500 0.26754500

Au -2.59800800 -0.84867700 1.15698900

Au 0.90250300 1.13192200 2.13892900

Au 2.61654800 -0.88997600 1.16608200

Au 0.14171200 1.38425300 -1.70709900

Au -3.26157200 -1.95406400 -1.36149400

Au 1.31063300 -2.88322600 -0.24064600

Au 4.48064700 -0.33123900 -0.80836200

Au -0.60948600 -1.29681500 -1.77201400

Au 2.62991100 1.63661000 -0.12173200

Au -1.78839100 3.39020900 -1.15701200

Au -1.84472000 1.99343800 1.27585000

Au -2.55136800 0.72583900 -1.19456200

Mo -1.37408600 0.25950400 3.15471200

***Endohedral Tc@Au_16_^–^ (a~f)***

**TcAu_16_^–^-*a***

Au 0.00000000 3.96370300 -2.69660200

Au 0.00000000 1.34496800 -3.32533900

Au 0.00000000 2.38209200 -0.52025700

Au -2.29848400 -1.45645100 0.94715400

Au -1.46174600 0.00000000 3.31768100

Au 0.00000000 -2.37720300 2.39993600

Au 0.00000000 -2.38209200 -0.52025700

Au 1.52867200 0.00000000 -1.35175100

Au 2.29848400 -1.45645100 0.94715400

Au 0.00000000 -1.34496800 -3.32533900

Au 2.29848400 1.45645100 0.94715400

Au 1.46174600 0.00000000 3.31768100

Au -1.52867200 0.00000000 -1.35175100

Au 0.00000000 -3.96370300 -2.69660200

Au 0.00000000 2.37720300 2.39993600

Au -2.29848400 1.45645100 0.94715400

Tc 0.00000000 0.00000000 1.03627700

**TcAu_16_^–^-*b***

Au 1.68510400 1.86343300 1.94394000

Au 0.31777100 -2.96378300 0.09889700

Au 0.23206900 -1.64196800 2.60472900

Au -2.80466000 1.34443100 0.37647300

Au -1.84054100 -1.29510800 0.68636300

Au -0.88119800 0.83944000 2.45693000

Au 2.68899400 -1.75794100 1.43287600

Au -0.62497700 2.91669300 0.62137800

Au -2.78405500 -0.42631400 -1.87388000

Au -0.26366300 -1.37950800 -2.17395700

Au -0.76754200 1.46250200 -1.79744300

Au 1.78746600 0.54691600 -2.54630800

Au 1.67471200 2.66566200 -0.81476300

Au 3.34419900 0.54433400 0.02140400

Au 2.41143400 -1.85941200 -1.36904800

Au -4.54144700 -0.81147400 0.21859900

Tc 0.67303000 -0.08801100 0.20909000

**TcAu_16_^–^-*c***

Au -2.56378100 -1.78183800 1.16960900

Au -2.63814500 1.11719400 1.32549200

Au -0.89153400 -0.11464200 -2.80982300

Au -0.74657500 -0.45775200 2.81589000

Au 1.16067700 1.43708500 -1.79337700

Au 1.19314100 -1.69912700 -1.81461200

Au -3.13172200 -0.17430700 -1.14198000

Au 2.80424100 -2.71871700 0.25256600

Au 3.18397100 -0.15155900 -0.71523300

Au 0.50574100 3.85034200 -0.25942600

Au 1.82037200 -0.41337200 1.77805400

Au -0.08182900 2.07313400 1.83337700

Au 0.16238800 -2.64807000 1.09207700

Au -1.41303700 -2.49982400 -1.30800700

Au -1.53036900 2.18218500 -1.11031900

Au 2.41809600 2.08005700 0.68347300

Tc -0.46230600 -0.14842500 0.00411400

**TcAu_16_^–^-*d***

Au 0.63706400 2.27741500 1.55457200

Au 2.22434200 -0.05271200 2.32084700

Au -2.21736100 -1.32013500 1.40896100

Au -0.39412100 0.02064900 3.02697500

Au 0.53072700 -2.30816200 1.60087500

Au 2.76375900 1.35575700 0.00000000

Au -1.41223300 -3.55882000 0.00000000

Au -2.15769000 1.42338600 -1.40510200

Au 0.63706400 2.27741500 -1.55457200

Au -2.21736100 -1.32013500 -1.40896100

Au -0.39412100 0.02064900 -3.02697500

Au 0.53072700 -2.30816200 -1.60087500

Au 2.66845900 -1.50659100 0.00000000

Au 2.22434200 -0.05271200 -2.32084700

Au -2.15769000 1.42338600 1.40510200

Au -1.28235200 3.63802500 0.00000000

Tc 0.03021300 -0.01700200 0.00000000

**TcAu_16_^–^-*e***

Au 1.93336000 2.25990400 0.81389800

Au -0.82255400 -2.28437500 1.42013500

Au -0.38686700 -0.04110400 3.08089700

Au -2.27086700 1.43661700 -1.01391600

Au -2.55824100 -0.02128700 1.44517300

Au -0.82370800 2.24403700 1.47927900

Au 2.24073400 -0.03302900 2.45998200

Au 0.01120000 2.81425300 -1.15063200

Au -2.27112100 -1.40797000 -1.05415400

Au 0.01071700 -2.78179200 -1.22459400

Au -0.31206100 0.03503000 -2.54128800

Au 2.13599900 -1.35969600 -2.06435100

Au 2.13579600 1.41463500 -2.02811400

Au 3.44362300 0.00119400 -0.02084400

Au 1.93456900 -2.28109400 0.75299200

Au -4.56392200 0.00655300 -0.45000500

Tc 0.30009700 -0.00344500 0.17553500

**TcAu_16_^–^-*f***

Au 0.41051100 -2.27300100 0.04902500

Au -2.11749400 -2.36647000 1.41938600

Au 0.40829500 1.17885300 1.94385900

Au 0.16000400 -1.53510400 2.79555300

Au -2.11928700 0.07056500 2.75661900

Au -2.11791400 2.41287000 1.33688200

Au 2.38530800 1.65959600 -0.03378300

Au 2.38622000 -0.86019900 -1.41800400

Au 4.56478300 -0.00059600 0.00180400

Au 0.16170100 3.19034500 -0.06806400

Au 0.41100700 1.09448600 -1.99136400

Au -2.11555700 2.35341200 -1.44161400

Au -2.11515300 -0.04703100 -2.75942700

Au 0.16360400 -1.65391300 -2.72781900

Au 2.38474800 -0.79841700 1.45614300

Au -2.11582900 -2.42456900 -1.31858300

Tc -1.35024900 -0.00152100 -0.00112300

***Plane structures* *TcAu_16_*^–^ (*g*~*i*)**

**TcAu_16_^–^-*g***

Au 0.00000000 2.74839600 -4.17827700

Au 0.00000000 4.10312700 -1.84299800

Au 0.00000000 5.45431700 0.47189700

Au 0.00000000 0.00000000 -4.16894100

Au 0.00000000 1.35338400 -1.76909500

Au 0.00000000 2.70660000 0.62289800

Au 0.00000000 4.14278800 2.92094700

Au 0.00000000 -2.74839600 -4.17827700

Au 0.00000000 -1.35338400 -1.76909500

Au 0.00000000 1.39499900 3.02744800

Au 0.00000000 -4.10312700 -1.84299800

Au 0.00000000 -2.70660000 0.62289800

Au 0.00000000 -1.39499900 3.02744800

Au 0.00000000 -5.45431700 0.47189700

Au 0.00000000 -4.14278800 2.92094700

Tc 0.00000000 0.00000000 0.61617200

Au 0.00000000 0.00000000 5.32791300

**TcAu_16_^–^-*h***

Au -3.94306300 -2.96389600 0.00000000

Au -4.65177000 -0.33754200 0.00000000

Au -5.23787800 2.31854900 0.00000000

Au -1.33989300 -3.74514800 0.00000000

Au -1.97548000 -1.05130300 0.00000000

Au -2.61038400 1.58118800 0.00000000

Au -3.27458800 4.21817500 0.00000000

Au 1.29632300 -4.49507300 0.00000000

Au 0.67270200 -1.83363900 0.00000000

Au 0.00000000 0.81742800 0.00000000

Au -0.60791400 3.52604200 0.00000000

Au 3.30538400 -2.66718200 0.00000000

Au 2.63844500 0.06737300 0.00000000

Au 1.98268100 2.74658400 0.00000000

Au 5.25598900 -0.79547900 0.00000000

Au 4.62020300 2.01386600 0.00000000

Tc 7.10860700 1.10243200 0.00000000

**TcAu_16_^–^-*i***

Au 0.00000000 2.73028400 4.05061500

Au 0.00000000 4.14519700 1.71126900

Au 0.00000000 5.44802100 -0.66395900

Au 0.00000000 0.00000000 4.03508100

Au 0.00000000 1.39091200 1.65481600

Au 0.00000000 2.70608300 -0.71355500

Au 0.00000000 4.10220400 -3.06396100

Au 0.00000000 -2.73028400 4.05061500

Au 0.00000000 -1.39091200 1.65481600

Au 0.00000000 0.00000000 -0.71171100

Au 0.00000000 1.38789500 -3.16219700

Au 0.00000000 -4.14519700 1.71126900

Au 0.00000000 -2.70608300 -0.71355500

Au 0.00000000 -1.38789500 -3.16219700

Au 0.00000000 -5.44802100 -0.66395900

Au 0.00000000 -4.10220400 -3.06396100

Tc 0.00000000 0.00000000 -5.41870700

***Exohedral TcAu_16_^–^ (j~k)***

**TcAu_16_^–^-*j***

Au 0.60614800 3.21126500 0.42288100

Au 0.06534600 -1.63774200 2.06316500

Au 1.94208500 -0.70864600 -1.79622100

Au -1.53388700 -3.08658800 0.31088200

Au -2.53290600 -0.67735300 1.24169000

Au 1.03593400 1.07462400 2.13273600

Au 2.55864600 -1.02632700 1.15011200

Au 0.18944400 1.40441000 -1.80220000

Au -3.31919900 -1.80345300 -1.35900700

Au 1.11716200 -2.91171800 -0.33734000

Au 4.45778400 -0.46645700 -0.73940400

Au -0.68643600 -1.27980100 -1.85139600

Au 2.61169700 1.53011400 -0.23516400

Au -1.62636200 3.41011800 -1.19117200

Au -1.71452600 1.97583200 1.31396900

Au -2.51520800 0.81398200 -1.17853800

Tc -1.20469900 0.32654800 3.40803400

**TcAu_16_^–^-*k***

Au -0.77699400 1.57495800 2.09353800

Au -0.12787200 -2.15296700 -2.29245800

Au 1.09941900 1.86055800 0.00000000

Au -0.23603400 -0.13320200 -4.21065500

Au -2.50089100 -0.60425500 -2.36989600

Au -0.12787200 -2.15296700 2.29245800

Au -2.59173000 0.76991900 0.00000000

Au 1.63422000 0.03288500 -2.20260700

Au -0.77699400 1.57495800 -2.09353800

Au -0.23603400 -0.13320200 4.21065500

Au 5.43356500 0.48835900 0.00000000

Au -1.59441400 -1.85823000 0.00000000

Au -1.31594300 3.27359600 0.00000000

Au 1.36738800 -1.99275700 0.00000000

Au -2.50089100 -0.60425500 2.36989600

Au 1.63422000 0.03288500 2.20260700

Tc 2.97050000 0.04357500 0.00000000
